# 3,5-Bis(4-bromo­phen­yl)-1-phenyl-4,5-dihydro-1*H*-pyrazole

**DOI:** 10.1107/S1600536810015795

**Published:** 2010-05-08

**Authors:** S. Samshuddin, B. Narayana, H. S. Yathirajan, A. P. Safwan, Edward R. T. Tiekink

**Affiliations:** a Department of Studies in Chemistry, Mangalore University, Mangalagangotri 574 199, India; b Department of Studies in Chemistry, University of Mysore, Manasagangotri, Mysore 570 006, India; c Department of Chemistry, University of Malaya, 50603 Kuala Lumpur, Malaysia

## Abstract

In the title compound, C_21_H_16_Br_2_N_2_, the central pyrazole ring adopts an flattened envelope conformation, with the stereogenic C atom in the flap position. The deviations from planarity for this ring are relatively minor (r.m.s. deviation = 0.045 Å) and the dihedral angles formed with the N- and C_imine_-bound benzene rings are 7.73 (13) and 11.00 (13)°, respectively. By contrast, the benzene ring bound at the chiral C atom is almost orthogonal to the rest of the mol­ecule; the dihedral angle formed between this ring and the pyrazole ring is 79.53 (13)°. In the crystal, the packing is stabilized by C—H⋯N and C—H⋯Br inter­actions.

## Related literature

For the pharmacological activity of pyrazoline derivatives, see: Hes *et al.* (1978 ▶); Amir *et al.* (2008 ▶); Sarojini *et al.* (2010 ▶). For related structures, see: Fun *et al.* (2010 ▶); Yathirajan *et al.* (2007 ▶). For the structure of the parent compound, 1,3,5-triphenyl-2-pyrazoline, see: Foces-Foces *et al.* (2001 ▶). For conformational analysis, see: Cremer & Pople (1975 ▶).
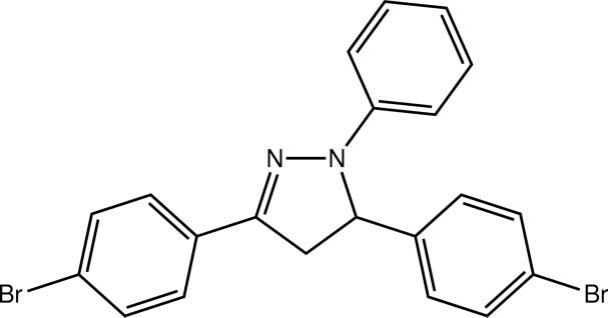


## Experimental

### 

#### Crystal data

C_21_H_16_Br_2_N_2_
*M* *_r_* = 456.18
Orthorhombic, 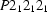

*a* = 10.5815 (3) Å
*b* = 11.2119 (3) Å
*c* = 15.4569 (4) Å
*V* = 1833.79 (9) Å^3^
*Z* = 4
Mo *K*α radiation
μ = 4.43 mm^−1^
*T* = 100 K
0.35 × 0.15 × 0.08 mm

#### Data collection

Bruker SMART APEX diffractometer
Absorption correction: multi-scan (*SADABS*; Sheldrick, 1996 ▶) *T*_min_ = 0.537, *T*_max_ = 0.746
17504 measured reflections
4201 independent reflections
3753 reflections with *I* > 2σ(*I*)
*R*_int_ = 0.044

#### Refinement

*R*[*F* ^2^ > 2σ(*F* ^2^)] = 0.027
*wR*(*F* ^2^) = 0.052
*S* = 1.02
4201 reflections
226 parameters
H-atom parameters constrained
Δρ_max_ = 0.60 e Å^−3^
Δρ_min_ = −0.40 e Å^−3^
Absolute structure: Flack (1983 ▶), 1804 Friedel pairs
Flack parameter: 0.003 (7)

### 

Data collection: *APEX2* (Bruker, 2008 ▶); cell refinement: *SAINT* (Bruker, 2008 ▶); data reduction: *SAINT*; program(s) used to solve structure: *SIR97* (Altomare *et al.*, 1999 ▶); program(s) used to refine structure: *SHELXL97* (Sheldrick, 2008 ▶); molecular graphics: *ORTEP-3* (Farrugia, 1997 ▶) and *DIAMOND* (Brandenburg, 2006 ▶); software used to prepare material for publication: *publCIF* (Westrip, 2010 ▶).

## Supplementary Material

Crystal structure: contains datablocks global, I. DOI: 10.1107/S1600536810015795/hb5428sup1.cif

Structure factors: contains datablocks I. DOI: 10.1107/S1600536810015795/hb5428Isup2.hkl

Additional supplementary materials:  crystallographic information; 3D view; checkCIF report

## Figures and Tables

**Table 1:** Hydrogen-bond geometry (Å, °)

*D*—H⋯*A*	*D*—H	H⋯*A*	*D*⋯*A*	*D*—H⋯*A*
C5—H5⋯N1^i^	0.95	2.58	3.374 (3)	141
C20—H20⋯Br1^ii^	0.95	2.92	3.768 (2)	148

Symmetry codes: (i) 
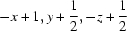
; (ii) 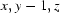.

**Fig. 1.**
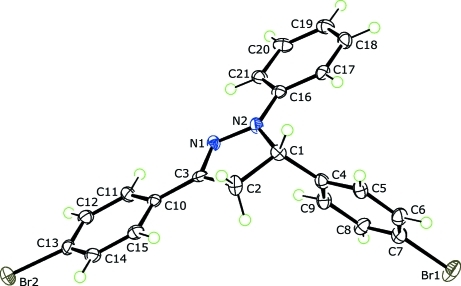
The molecular structure of (I) showing displacement ellipsoids at the 50% probability level.

**Fig. 2.**
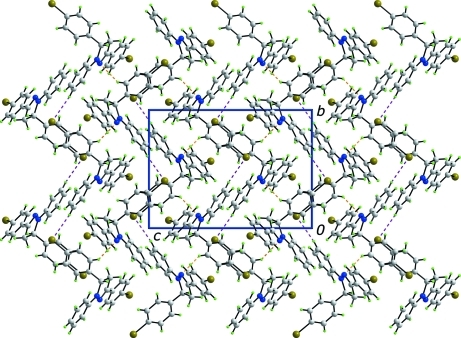
A view in projection down the a axis of the crystal packing in (I) mediated by C–H···N and C–H···Br contacts, shown as orange and purple dashed lines, respectively.

## supplementary crystallographic information

### Comment

Derivatives of pyrazoline possess a range of pharmacological activities, having, for example, anti-tumour, anti-microbial, and anti-tubercular activities (Hes *et al.*, 1978; Amir *et al.*, 2008). Further, some of these compounds also have anti-inflammatory, anti-diabetic, anaesthetic, analgesic and DPPH scavenging properties (Sarojini *et al.*, 2010). In continuation of previous structural studies of pyrazoline derivatives (Fun *et al.*, 2010, Yathirajan *et al.*, 2007), the title compound, (I), was synthesised and its crystal structure determined.

The structure analysis of (I) shows the C1 centre to have an *S* configuration, Fig. 1. The conformation of the central pyrazole ring is an envelope on the C1 atom as defined by the ring-puckering parameters of q_2_ = 0.101 (3) Å and φ_2_ = 251.7 (14) ° (Cremer & Pople, 1975). That being stated, the maximum deviations from the five atoms of the ring are 0.052 (2) and -0.064 (3) Å for the N2 and C1 atoms, respectively; the r.m.s. deviation = 0.0450 Å. The N2- and C3-bound benzene rings are approximately co-planar with the central ring as seen in the dihedral angles formed between their respective least-squares planes and that through the pyrazole ring of 7.73 (13) and 11.00 (13) °; the dihedral angle between these benzene rings is 4.32 (12) °. By contrast, the C1-bound benzene ring is almost orthogonal to the remaining molecule with a dihedral angle of 79.53 (13) ° formed between it and the pyrazole ring. To a first approximation, the overall conformation in (I) resembles that in the analogous 1,3,5-triphenyl-2-pyrazoline "parent" compound although the deviations from planarity are slightly greater in the literature structure (Foces-Foces *et al.*, 2001). Further, the N1–N2 [1.369 (3) Å] and N1═C3 [1.291 (3) Å] bond distances in (I) are comparable to the equivalent distances in 1,3,5-triphenyl-2-pyrazoline of 1.387 (5) and 1.285 (7) Å, respectively.

The molecules are consolidated into a 3-D network by C–H···N and C—H···Br contacts, Fig. 2 and Table 1.

### Experimental

A mixture of (2*E*)-1,3-bis(4-bromophenyl)prop-2-en-1-one (3.66 g, 0.01 mol) and phenyl hydrazine (1.08 g, 0.01 mol) in glacial acetic acid (50 ml) was refluxed for 6 h. The reaction mixture was cooled and poured into ice-cold water (50 ml). The precipitate was collected by filtration and purified by recrystallization from ethanol. Yellow blocks of (I) were grown from toluene by slow evaporation; the yield was 86%, m.pt. 481 K. Analytical data (%): Found (Calc'd): C 55.21 (55.29); H 3.48 (3.54); N 6.10 (6.14).

### Refinement

Carbon-bound H-atoms were placed in calculated positions (C—H 0.95 to 1.00 Å) and were included in the refinement in the riding model approximation, with *U*_iso_(H) set to 1.2 to 1.5*U*_eq_(C).

### Crystal data

**Table:**

C_21_H_16_Br_2_N_2_	*F*(000) = 904
*M**_r_* = 456.18	*D*_x_ = 1.652 Mg m^−^^3^
Orthorhombic, *P*2_1_2_1_2_1_	Mo *K*α radiation, λ = 0.71073 Å
Hall symbol: P 2ac 2ab	Cell parameters from 4444 reflections
*a* = 10.5815 (3) Å	θ = 2.3–26.2°
*b* = 11.2119 (3) Å	µ = 4.43 mm^−^^1^
*c* = 15.4569 (4) Å	*T* = 100 K
*V* = 1833.79 (9) Å^3^	Block, yellow
*Z* = 4	0.35 × 0.15 × 0.08 mm

### Data collection

**Table:**

Bruker SMART APEX diffractometer	4201 independent reflections
Radiation source: fine-focus sealed tube	3753 reflections with *I* > 2σ(*I*)
graphite	*R*_int_ = 0.044
ω scans	θ_max_ = 27.5°, θ_min_ = 2.2°
Absorption correction: multi-scan (*SADABS*; Sheldrick, 1996)	*h* = −13→13
*T*_min_ = 0.537, *T*_max_ = 0.746	*k* = −14→14
17504 measured reflections	*l* = −20→20

### Refinement

**Table:**

Refinement on *F*^2^	Secondary atom site location: difference Fourier map
Least-squares matrix: full	Hydrogen site location: inferred from neighbouring sites
*R*[*F*^2^ > 2σ(*F*^2^)] = 0.027	H-atom parameters constrained
*wR*(*F*^2^) = 0.052	*w* = 1/[σ^2^(*F*_o_^2^) + (0.0105*P*)^2^] where *P* = (*F*_o_^2^ + 2*F*_c_^2^)/3
*S* = 1.02	(Δ/σ)_max_ < 0.001
4201 reflections	Δρ_max_ = 0.60 e Å^−^^3^
226 parameters	Δρ_min_ = −0.40 e Å^−^^3^
0 restraints	Absolute structure: Flack (1983), 1804 Friedel pairs
Primary atom site location: structure-invariant direct methods	Flack parameter: 0.003 (7)

### Special details

**Table:**

Geometry. All s.u.'s (except the s.u. in the dihedral angle between two l.s. planes) are estimated using the full covariance matrix. The cell s.u.'s are taken into account individually in the estimation of s.u.'s in distances, angles and torsion angles; correlations between s.u.'s in cell parameters are only used when they are defined by crystal symmetry. An approximate (isotropic) treatment of cell s.u.'s is used for estimating s.u.'s involving l.s. planes.
Refinement. Refinement of *F*^2^ against ALL reflections. The weighted *R*-factor wR and goodness of fit *S* are based on *F*^2^, conventional *R*-factors *R* are based on *F*, with *F* set to zero for negative *F*^2^. The threshold expression of *F*^2^ > 2σ(*F*^2^) is used only for calculating *R*-factors(gt) etc. and is not relevant to the choice of reflections for refinement. *R*-factors based on *F*^2^ are statistically about twice as large as those based on *F*, and *R*- factors based on ALL data will be even larger.

### Fractional atomic coordinates and isotropic or equivalent isotropic displacement parameters (Å^2^)

**Table:**

	*x*	*y*	*z*	*U*_iso_*/*U*_eq_	
Br1	0.45694 (3)	0.39983 (2)	−0.095975 (18)	0.03237 (9)	
Br2	1.28094 (3)	−0.06644 (3)	0.379191 (19)	0.02784 (8)	
N1	0.68637 (18)	−0.10495 (19)	0.19763 (12)	0.0155 (5)	
N2	0.56377 (19)	−0.07418 (19)	0.18013 (13)	0.0175 (5)	
C1	0.5321 (3)	0.0486 (2)	0.20739 (15)	0.0184 (5)	
H1	0.4556	0.0479	0.2453	0.022*	
C2	0.6507 (2)	0.0809 (3)	0.26139 (16)	0.0226 (6)	
H2A	0.6878	0.1576	0.2422	0.027*	
H2B	0.6304	0.0859	0.3238	0.027*	
C3	0.7385 (2)	−0.0217 (2)	0.24286 (16)	0.0144 (6)	
C4	0.5117 (2)	0.1321 (2)	0.13185 (17)	0.0165 (6)	
C5	0.4266 (3)	0.2261 (2)	0.13916 (17)	0.0206 (6)	
H5	0.3790	0.2351	0.1909	0.025*	
C6	0.4100 (3)	0.3064 (2)	0.07249 (17)	0.0230 (6)	
H6	0.3521	0.3707	0.0781	0.028*	
C7	0.4791 (3)	0.2917 (2)	−0.00225 (17)	0.0208 (6)	
C8	0.5631 (3)	0.1994 (2)	−0.01219 (16)	0.0213 (6)	
H8	0.6087	0.1901	−0.0647	0.026*	
C9	0.5803 (2)	0.1200 (2)	0.05566 (17)	0.0193 (6)	
H9	0.6394	0.0567	0.0500	0.023*	
C10	0.8685 (2)	−0.0309 (2)	0.27394 (16)	0.0141 (6)	
C11	0.9485 (3)	−0.1218 (2)	0.24481 (16)	0.0179 (6)	
H11	0.9182	−0.1784	0.2041	0.021*	
C12	1.0714 (3)	−0.1300 (2)	0.27476 (17)	0.0199 (6)	
H12	1.1256	−0.1914	0.2544	0.024*	
C13	1.1146 (2)	−0.0478 (3)	0.33476 (17)	0.0190 (6)	
C14	1.0378 (2)	0.0425 (2)	0.36482 (16)	0.0190 (5)	
H14	1.0685	0.0981	0.4062	0.023*	
C15	0.9159 (2)	0.0512 (2)	0.33413 (16)	0.0178 (6)	
H15	0.8631	0.1140	0.3541	0.021*	
C16	0.4888 (2)	−0.1457 (2)	0.12849 (16)	0.0168 (5)	
C17	0.3627 (2)	−0.1161 (2)	0.11246 (16)	0.0195 (6)	
H17	0.3271	−0.0468	0.1380	0.023*	
C18	0.2893 (3)	−0.1876 (3)	0.05953 (17)	0.0223 (6)	
H18	0.2036	−0.1666	0.0494	0.027*	
C19	0.3379 (3)	−0.2884 (3)	0.02115 (17)	0.0222 (6)	
H19	0.2869	−0.3362	−0.0157	0.027*	
C20	0.4627 (3)	−0.3189 (2)	0.03732 (17)	0.0225 (6)	
H20	0.4969	−0.3886	0.0113	0.027*	
C21	0.5387 (3)	−0.2497 (2)	0.09078 (16)	0.0188 (5)	
H21	0.6236	−0.2725	0.1018	0.023*	

### Atomic displacement parameters (Å^2^)

**Table:**

	*U*^11^	*U*^22^	*U*^33^	*U*^12^	*U*^13^	*U*^23^
Br1	0.0559 (2)	0.01873 (14)	0.02246 (14)	0.00349 (14)	−0.01182 (14)	0.00164 (12)
Br2	0.01697 (13)	0.03195 (17)	0.03461 (17)	0.00011 (12)	−0.00644 (13)	0.00337 (14)
N1	0.0142 (11)	0.0171 (11)	0.0151 (10)	−0.0003 (9)	−0.0004 (8)	0.0027 (10)
N2	0.0142 (11)	0.0189 (12)	0.0193 (10)	0.0029 (10)	−0.0035 (9)	−0.0018 (9)
C1	0.0191 (14)	0.0189 (13)	0.0172 (13)	0.0012 (12)	0.0014 (11)	−0.0035 (11)
C2	0.0227 (14)	0.0244 (16)	0.0208 (13)	0.0058 (13)	−0.0072 (12)	−0.0030 (13)
C3	0.0172 (14)	0.0140 (13)	0.0121 (12)	−0.0011 (10)	0.0006 (10)	0.0028 (10)
C4	0.0134 (13)	0.0182 (13)	0.0179 (13)	−0.0005 (10)	−0.0044 (11)	−0.0022 (11)
C5	0.0194 (14)	0.0231 (14)	0.0195 (14)	0.0009 (11)	−0.0028 (11)	−0.0035 (12)
C6	0.0255 (16)	0.0166 (14)	0.0269 (16)	0.0043 (12)	−0.0055 (13)	−0.0047 (12)
C7	0.0284 (17)	0.0171 (14)	0.0170 (13)	−0.0012 (12)	−0.0115 (12)	−0.0024 (12)
C8	0.0264 (17)	0.0217 (15)	0.0159 (13)	−0.0010 (12)	−0.0008 (12)	−0.0018 (11)
C9	0.0176 (14)	0.0183 (15)	0.0219 (13)	0.0058 (11)	−0.0012 (11)	−0.0030 (12)
C10	0.0150 (14)	0.0137 (13)	0.0136 (12)	−0.0018 (10)	0.0004 (10)	0.0022 (10)
C11	0.0209 (15)	0.0173 (14)	0.0156 (12)	−0.0048 (12)	−0.0016 (12)	−0.0017 (10)
C12	0.0203 (15)	0.0163 (14)	0.0232 (14)	0.0003 (11)	0.0030 (12)	0.0011 (11)
C13	0.0124 (13)	0.0239 (15)	0.0205 (13)	−0.0005 (11)	−0.0010 (11)	0.0094 (12)
C14	0.0222 (14)	0.0176 (13)	0.0173 (13)	−0.0044 (11)	−0.0033 (12)	0.0025 (11)
C15	0.0210 (14)	0.0143 (13)	0.0180 (13)	0.0003 (11)	0.0037 (11)	0.0002 (11)
C16	0.0189 (13)	0.0188 (12)	0.0126 (12)	−0.0049 (10)	0.0008 (11)	0.0013 (11)
C17	0.0218 (14)	0.0186 (13)	0.0181 (14)	−0.0005 (11)	−0.0008 (12)	0.0025 (12)
C18	0.0187 (15)	0.0292 (16)	0.0190 (14)	−0.0032 (13)	−0.0030 (13)	0.0058 (12)
C19	0.0255 (16)	0.0243 (16)	0.0168 (14)	−0.0098 (13)	−0.0023 (12)	0.0001 (12)
C20	0.0249 (16)	0.0230 (14)	0.0196 (14)	−0.0061 (13)	0.0040 (13)	−0.0033 (11)
C21	0.0159 (14)	0.0221 (13)	0.0183 (13)	−0.0028 (11)	0.0021 (12)	0.0021 (12)

### Geometric parameters (Å, °)

**Table:**

Br1—C7	1.903 (3)	C9—H9	0.9500
Br2—C13	1.901 (2)	C10—C11	1.399 (4)
N1—C3	1.291 (3)	C10—C15	1.402 (4)
N1—N2	1.369 (3)	C11—C12	1.383 (4)
N2—C16	1.382 (3)	C11—H11	0.9500
N2—C1	1.478 (3)	C12—C13	1.385 (4)
C1—C4	1.512 (4)	C12—H12	0.9500
C1—C2	1.550 (4)	C13—C14	1.379 (4)
C1—H1	1.0000	C14—C15	1.377 (4)
C2—C3	1.506 (4)	C14—H14	0.9500
C2—H2A	0.9900	C15—H15	0.9500
C2—H2B	0.9900	C16—C17	1.397 (3)
C3—C10	1.461 (4)	C16—C21	1.407 (3)
C4—C9	1.390 (4)	C17—C18	1.383 (4)
C4—C5	1.391 (4)	C17—H17	0.9500
C5—C6	1.380 (4)	C18—C19	1.377 (4)
C5—H5	0.9500	C18—H18	0.9500
C6—C7	1.377 (4)	C19—C20	1.386 (4)
C6—H6	0.9500	C19—H19	0.9500
C7—C8	1.373 (4)	C20—C21	1.390 (4)
C8—C9	1.388 (4)	C20—H20	0.9500
C8—H8	0.9500	C21—H21	0.9500
			
C3—N1—N2	109.2 (2)	C11—C10—C15	118.4 (2)
N1—N2—C16	120.8 (2)	C11—C10—C3	121.0 (2)
N1—N2—C1	113.1 (2)	C15—C10—C3	120.6 (2)
C16—N2—C1	125.1 (2)	C12—C11—C10	120.6 (2)
N2—C1—C4	112.9 (2)	C12—C11—H11	119.7
N2—C1—C2	100.8 (2)	C10—C11—H11	119.7
C4—C1—C2	112.8 (2)	C11—C12—C13	119.3 (2)
N2—C1—H1	110.0	C11—C12—H12	120.3
C4—C1—H1	110.0	C13—C12—H12	120.3
C2—C1—H1	110.0	C14—C13—C12	121.3 (2)
C3—C2—C1	102.6 (2)	C14—C13—Br2	120.3 (2)
C3—C2—H2A	111.2	C12—C13—Br2	118.3 (2)
C1—C2—H2A	111.2	C15—C14—C13	119.2 (2)
C3—C2—H2B	111.2	C15—C14—H14	120.4
C1—C2—H2B	111.2	C13—C14—H14	120.4
H2A—C2—H2B	109.2	C14—C15—C10	121.1 (2)
N1—C3—C10	122.0 (2)	C14—C15—H15	119.4
N1—C3—C2	113.1 (2)	C10—C15—H15	119.4
C10—C3—C2	124.9 (2)	N2—C16—C17	120.9 (2)
C9—C4—C5	118.8 (2)	N2—C16—C21	120.3 (2)
C9—C4—C1	121.3 (2)	C17—C16—C21	118.8 (2)
C5—C4—C1	119.9 (2)	C18—C17—C16	120.3 (3)
C6—C5—C4	121.0 (2)	C18—C17—H17	119.9
C6—C5—H5	119.5	C16—C17—H17	119.9
C4—C5—H5	119.5	C19—C18—C17	121.4 (3)
C7—C6—C5	118.8 (2)	C19—C18—H18	119.3
C7—C6—H6	120.6	C17—C18—H18	119.3
C5—C6—H6	120.6	C18—C19—C20	118.7 (3)
C8—C7—C6	121.8 (2)	C18—C19—H19	120.6
C8—C7—Br1	118.3 (2)	C20—C19—H19	120.6
C6—C7—Br1	119.8 (2)	C19—C20—C21	121.4 (3)
C7—C8—C9	118.9 (2)	C19—C20—H20	119.3
C7—C8—H8	120.5	C21—C20—H20	119.3
C9—C8—H8	120.5	C20—C21—C16	119.5 (3)
C8—C9—C4	120.6 (2)	C20—C21—H21	120.3
C8—C9—H9	119.7	C16—C21—H21	120.3
C4—C9—H9	119.7		
			
C3—N1—N2—C16	176.5 (2)	N1—C3—C10—C11	10.1 (4)
C3—N1—N2—C1	7.3 (3)	C2—C3—C10—C11	−171.9 (2)
N1—N2—C1—C4	110.1 (2)	N1—C3—C10—C15	−169.7 (2)
C16—N2—C1—C4	−58.6 (3)	C2—C3—C10—C15	8.4 (4)
N1—N2—C1—C2	−10.5 (3)	C15—C10—C11—C12	−0.1 (4)
C16—N2—C1—C2	−179.1 (2)	C3—C10—C11—C12	−179.8 (2)
N2—C1—C2—C3	9.2 (2)	C10—C11—C12—C13	0.6 (4)
C4—C1—C2—C3	−111.5 (2)	C11—C12—C13—C14	−0.4 (4)
N2—N1—C3—C10	178.0 (2)	C11—C12—C13—Br2	176.76 (19)
N2—N1—C3—C2	−0.3 (3)	C12—C13—C14—C15	−0.3 (4)
C1—C2—C3—N1	−6.1 (3)	Br2—C13—C14—C15	−177.42 (19)
C1—C2—C3—C10	175.7 (2)	C13—C14—C15—C10	0.8 (4)
N2—C1—C4—C9	−34.0 (3)	C11—C10—C15—C14	−0.7 (4)
C2—C1—C4—C9	79.5 (3)	C3—C10—C15—C14	179.1 (2)
N2—C1—C4—C5	148.6 (2)	N1—N2—C16—C17	178.2 (2)
C2—C1—C4—C5	−98.0 (3)	C1—N2—C16—C17	−13.9 (4)
C9—C4—C5—C6	−0.2 (4)	N1—N2—C16—C21	−1.9 (3)
C1—C4—C5—C6	177.3 (2)	C1—N2—C16—C21	165.9 (2)
C4—C5—C6—C7	0.4 (4)	N2—C16—C17—C18	179.0 (2)
C5—C6—C7—C8	0.2 (4)	C21—C16—C17—C18	−0.8 (4)
C5—C6—C7—Br1	179.2 (2)	C16—C17—C18—C19	−0.3 (4)
C6—C7—C8—C9	−1.1 (4)	C17—C18—C19—C20	0.9 (4)
Br1—C7—C8—C9	179.9 (2)	C18—C19—C20—C21	−0.3 (4)
C7—C8—C9—C4	1.3 (4)	C19—C20—C21—C16	−0.8 (4)
C5—C4—C9—C8	−0.6 (4)	N2—C16—C21—C20	−178.4 (2)
C1—C4—C9—C8	−178.1 (2)	C17—C16—C21—C20	1.4 (4)

### Hydrogen-bond geometry (Å, °)

**Table:**

*D*—H···*A*	*D*—H	H···*A*	*D*···*A*	*D*—H···*A*
C5—H5···N1^i^	0.95	2.58	3.374 (3)	141
C20—H20···Br1^ii^	0.95	2.92	3.768 (2)	148

Symmetry codes: (i) −*x*+1, *y*+1/2, −*z*+1/2; (ii) *x*, *y*−1, *z*.
